# Human cerebral cortex Cajal-Retzius neuron: development, structure and function. A Golgi study

**DOI:** 10.3389/fnana.2015.00021

**Published:** 2015-02-27

**Authors:** Miguel Marín-Padilla

**Affiliations:** The Geisel School of Medicine at DartmouthHanover, NH, USA

**Keywords:** cerebral cortex, Cajal-Retzius cell, development, structure, function

## Abstract

The development, morphology and possible functional activity of the Cajal-Retzius cell of the developing human cerebral cortex are explored herein. The C-RC, of extracortical origin, is the essential neuron of the neocortex first lamina. It receives inputs from afferent fibers that reach the first lamina early in development. Although the origin and function of these original afferent fibers remain unknown, their target is the first lamina sole neuron: the C-RC. This neuron orchestrates the arrival, size and stratification of all pyramidal neurons (of ependymal origin) of the neocortex gray matter. Its axonic terminals spread radially and horizontally throughout the entirety of the first lamina establishing contacts with the dendritic terminals of all gray matter pyramidal cells regardless of size, location and/or eventual functional roles. While the neuron axonic terminals spread radially and horizontally throughout the first lamina, the neuronal’ body undergoes progressive developmental dilution and locating any of them in the adult brain become quite difficult. The neuron bodies are probably retained in the older regions of the neocortex while their axonic collaterals will spread throughout its more recent ones and eventually will extend to great majority of the cortical surface. The neocortex first lamina evolution and composition and that of the C-RC are intertwined and mutually interdependent. It is not possible to understand the C-RC evolving morphology without understanding that of the first lamina. The first lamina composition and its structural and functional organizations obtained with different staining methods may be utterly different. These differences have added unnecessary confusion about its nature. The essential emptiness observed in hematoxylin and eosin preparations (most commonly used) contrast sharply with the concentration of dendrites (the cortex’ largest) obtained using special (MAP-2) stain for dendrites. Only Golgi preparations demonstrate the numerous dendritic and axonic terminals that compose the first lamina basic structure. High power microscopic views of Golgi preparations demonstrate the intimate anatomical and functional interrelationships among dendritic and axonic terminals as well as synaptic contacts between them. The C-RC’ essential morphology does not changes but it is progressively modified by the first lamina increase in thickness and in number of terminal dendrites and their subsequent maturation. This neuron variable morphologic appearance has been the source of controversy. Its morphology depends on the first lamina thickness that may be quite variable among different mammals. In rodents (most commonly used experimental mammal), the first lamina thickness, number and horizontal expansion of dendrites is but a fraction of those in humans. This differences are reflected in the C-RC’ morphology among mammals (including humans) and should not be thought as representing new types of neurons.

## Introduction

The so-called Cajal-Retzius (von Koelliker, 1996) of the neocortex first lamina has been the source of continued controversy since originally described by Cajal (1891) and (Retzius (1893, 1894)). Its morphology, types, biochemistry, persistence vs. disappearance and role continue to be debated (Cajal, 1911; Derer and Derer, 1990; Meyer and González-Hernández, 1993; Meyer et al., 1999; Soriano and Del Río, 2005; Myakhar et al., 2011; Gil-Sanz et al., 2013; Martínez-Cerdeño and Noctor, 2014).

Because of this neuron controversies have only contributed additional confusion, we still lack, more than a century of its original description, a clear understanding of this neuron nature, morphology and functional role. I will describe the human cerebral cortex Cajal-Retzius cell developmental morphology and that of the first lamina, which I consider to be inseparable and mutually codependent.

Most descriptions about this neuron have focused on its variable morphology, although few explored the reasons why is that. What is a C-RC? Why is it morphology variable? Does it persist or does it disappear? How many first lamina neurons deserve the designation of C-RC? What is its functional target? What is its interrelationship to the neocortex first lamina? It is present in the adult brain? And, more important, what is its functional role in the developing vs. the adult brain? These and other questions about this essential neuron of the neocortex first lamina are explored herein using the rapid Golgi procedure.

The paper honors Camilo Golgi “reazione near” (Golgi, 1873) and it countless contributions to Neurosciences and laments that nowadays it is seldom used remaining essentially ignored by young neuroscientists. Cajal improved the Golgi procedure, used it throughout his life and his contributions constitute the foundations of modern Neurosciences. His classic Golgi studies of the newborn cerebral cortex remain essential and unsurpassed (Cajal, 1899a,b, 1900, 1901).

## Observations

Concerning the C-RCs controversies the following commentaries may be pertinent. The neocortex first lamina thickness among mammals is quite variable and must be considered when describing and/or comparing the C-RC’ variable morphology among them. In most mammals (specially in rodents) the first lamina thickness, the number of pyramidal neurons reaching it and the functional maturation of their terminal dendritic branches represent but small fractions of those observed in the human brain. These differences will be reflected in the structure and thickness of both the first lamina and the C-RCs for each mammalian species. While the C-RC morphology among mammals may be quite variable, it could simply reflect developmental adaptations and should not be thought as representing different types of neurons.

Moreover, the morphology of any C-RC depends on the plane of view. Originally, Cajal, Retzius and myself described three different morphologic types (pear shape, monopolar and bipolar) without realizing that they represented different views (planes of sections) of the same multipolar tangential neuron (Marín-Padilla, 1990). Therefore, any perpendicular view of the neuron body and dendrites represents an incomplete view of its actual three-dimensional morphology. Hence, single perpendicular views of this neuron’ body and dendrites could be quite variable without representing different types of neurons. On the other hand, the C-RCs axonic terminals should be similar in perpendicular and parallel sections of the first lamina. Therefore, perpendicular, parallel and tangentially cut sections of the cortex first lamina are required to understand this neuron three-dimensional morphology (Marín-Padilla, 1990). Comparison of human and other mammals’ data could be misleading and often erroneous.

The generally accepted idea that the C-RC represents a transient neuron that will eventually disappear is supported by the fact that it is quite difficult to visualize any of them in the adult brain. It must be understood that the difficulty only applies to the location the neuronal’ body, since its axonic terminals are recognized throughout the entire first lamina associated to the pyramidal cell dendrites (Marin-Padilla and Marin-Padilla, 1982; Marín-Padilla, 1984, 1990). Since the actual number C-RCs, is established early in development, the location of their body will undergoes progressive dilution during brain prenatal and postnatal developments. Since first recognized in 6-w-o (weeks of age) embryos, the human brain expansion is extraordinary as its surface area increases from 19 cm^2^ at 14-week-gestation to 700 cm^2^ at birth, to 1166 cm^2^ in the adult brain (Blinkov and Glezer, 1968). Moreover, the first lamina expansion throughout the cortex complex gyral patterns will be by far the greatest. The difficulty in locating a neuron body will increase exponentially during the brain extraordinary surface expansion. In the adult brain, the study of ten of thousand of sections would be necessary to localize a single C-RC body. During more than 50 years studying the human brain, I have identified C-RCs bodies, as well as their axonic terminals, throughout prenatal development, in many newborn infants, in few young children and, by serendipity, in a 4-year-old child, in a 22-year-old, a 72-year-old and a 86-year-old men and in a 36-year-old woman (Marín-Padilla, 1990, 2011; Martín et al., 1999). Considering the brain surface area and the relative small number of sections studied, these few observations will support the C-RCs’ persistence in the adult brain. A presume body of a C-RC has been identified in adult rats striate cortex of one hemisphere by injections of a retrograde label on the opposite one (Martínez-García et al., 1994). Others agree with the persistence of C-RCs in the adult brain (Meyer et al., 1999). A developmental “dilution” of the neuron body is a better explanation than their so-called disappearance in the adult brain.

A C-RC early function is the secretion of *Reelin* that will orchestrate the ascending migration of neuroblasts from the ependymal layer to the first lamina (using radial glia as guides), their transformation into pyramidal neurons, the order of migration, the neuron size and the eventual stratification of the gray matter (Marín-Padilla, 1992, 2014; Ogawa et al., 1995; Meyer et al., 1999; Soriano and Del Río, 2005). Since the number of pyramidal cells terminal dendrites reaching the first lamina increases progressively, the C-RC axonic terminal branches must elongate (*developmental horizontalization*) to reach all pyramidal cells throughout the cortex’ expanding surface, while the neuron body will remain on its original location. During late prenatal and postnatal cortical maturations, the dendrites undergo additional functional expansions compelling further extension (horizontalization) of C-RCs axonic terminals and, hence, increasing their body’s isolation and consequently, increasing the difficulty in locating any of them. While the C-RCs bodies may be difficult to locate in the adult brain, their long horizontal axonic terminals (Retzius tangential fibers) are recognized throughout the first lamina, in both perpendicular and tangentially cut sections (Marín-Padilla, 1984, 1990). In my opinion, the C-RC bodies are probably retained in the cortex older regions (primary motor, sensory, visual and acoustic cortices) while their axonic terminals continue to spread throughout more recent ones (associative regions) that eventually will represent the great majority of the cortex surface.

The first lamina has other types of neurons, mostly incorporated during late prenatal development. They have different morphologies, biochemical compositions and local functional roles and should not be confused with C-RCs. In my opinion, unique developmental, morphological and functional features characterize the C-RCs of the human developing and adult neocortex. C-RCs are also recognized in the cerebral cortex of amphibian and reptiles, attesting to their ancient participation in the cortex developmental structure and function (Cajal, 1911; Marín-Padilla, 1998).

It could be stated that most of the controversies about C-RCs do not seem to be applicable to the human cerebral cortex. Also that the neuron’ nature, development, morphology and possible function cannot be understood and/or separated from those of the neocortex first lamina.

### C-RC origin

The C-RC is the first neuron recognized in the developing neocortex of humans and other vertebrates (Marin-Padilla, 1971; Marín-Padilla, 1990, 1998, 2011). Its origin seems to be extracortical as other early subplate neurons (Marin-Padilla, 1971). C-RCs enter tangentially into developing cortex and are recognized under the pial surface as a large horizontal neuron. They are first recognized in 20-day-old cat and 6-week-old human embryos. Together with early pyramidal-like neurons and Martinotti cells, they constitute the mammals’ primordial cortical organization that resembles the primitive cortex of amphibian and reptiles (Marin-Padilla, 1971; Marín-Padilla, 1998). The subsequent incorporation of pyramidal neurons (of ependymal origin) establishes, simultaneously, the first lamina, above it, and the subplate zone below denoting the mammalian new cortex (neocortex) dual origin (Marin-Padilla, 1971; Marín-Padilla, 1998).

In humans, the ascending migration of pyramidal neurons toward the first lamina, start around 8-week-gestation and is nearly completed by 16-w-g (Marín-Padilla, 2011, 2014). At this age, all pyramidal neurons of the gray matter have terminal dendrites within the first lamina. The gray matter deeper and older pyramidal cells start their ascending functional maturation at this age. Concomitantly, the microvascularization, the first protoplasmic astrocytes and the first inhibitory neurons (of extracortical origin) are also recognized throughout the gray matter deeper and older region (Marín-Padilla, 2011, 2012). The pyramidal neuron is a mammalian innovation characterized by distinct developmental, morphological and functional features and by its permanent functional attachment to C-RCs axonic terminals and the first lamina (Marín-Padilla, 2014). C-RCs axonic terminals must elongate horizontally to contact all pyramidal cells terminal dendrites arriving to the first lamina throughout the developing cortex, while their bodies remain on their original location and become progressively “diluted”.

Neocortical neurons without these features, regardless of the pyramidal shape of their bodies, should not be considered and/or labeled as pyramidal neurons.

### C-RC developmental role

By secreting *Reelin*, the C-RC orchestrates the ascending migration, arrival, size and eventual stratification of the gray matter pyramidal neurons (Marín-Padilla, 1992, 2014). It establishes and maintains structural and functional interrelationships with the terminal dendrites of all pyramidal neurons reaching the first lamina. During late prenatal maturation of, the cortex, the terminal dendrites continue to expand adding new branches and the first lamina thickness increases accordingly as well as the horizontal spreading of C-RCs axonic terminals. The terminal dendrites functional growth and branching will continue during the neocortex postnatal maturation increasing the first lamina thickness, the further elongation C-RCs axonic terminals and the concomitant progressive “dilution” of their bodies (Marín-Padilla, 2011).

From the start of cortical development, afferent fibers without collaterals ascend from the white matter, reach the first lamina and become long horizontal fibers through its upper half (Cajal, 1911; Marin-Padilla, 1971; Marin-Padilla and Marin-Padilla, 1982). During early neocortical development, afferent fibers from the subplate reach the first lamina and hence the C-RCs (Marin-Padilla, 1971). Their function remains unknown, although a possible Gabaergic nature has been recently proposed (Myakhar et al., 2011). The C-RCs and these afferent fibers axonic terminals intermingle with pyramidal cells terminal dendrite throughout the first lamina. These afferent fibers functional target must also be the C-RCs since no other neurons are recognized in the first lamina during early development. It is possible that early subcortical centers of the developing brain could send inputs to the developing neocortex prior to the arrival of the more specific thalamic, callosal and cortico-cortical ones. The functional role of these early afferent fibers remains unknown.

### C-RC developmental morphology

**Figure 1:**
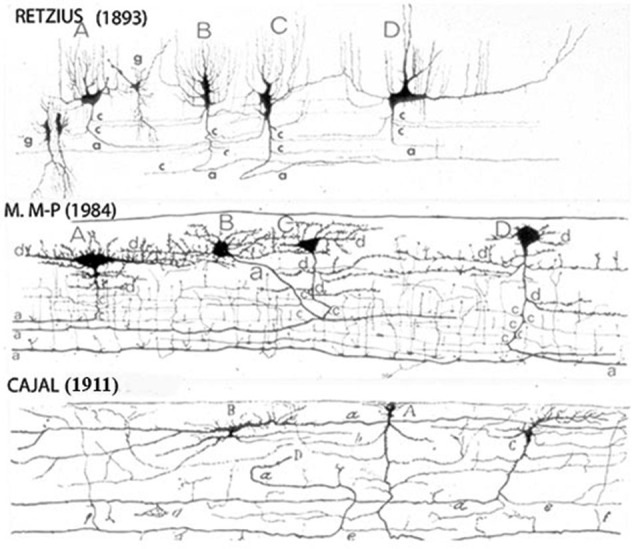
**Reproductions of Camera lucida drawings from the original Golgi studies of the human cerebral cortex of Retzius, Cajal and myself illustrating our agreement on the C-RCs morphological features**. The neuron’ body and dendrites are located in the lamina upper region and may be pear shape, monopolar and/or bipolar, its descending axon have long horizontal axonic collaterals through its middle region and its terminal axon becomes a long horizontal fiber through its lower region. Both, its axonic collaterals and terminal axon have numerous ascending and descending branchlets considered to represent the neuron functional outlets. The pyramidal cells terminal dendrites represent the first lamina main receptive elements. To reach all dendritic terminals, the C-RC axonic collaterals and terminal axonic fiber must elongate radially throughout the first lamina while its body remains on its original location and will undergo developmental dilution. The first lamina thickness in the newborn motor cortex is roughly 200 micrometers. (From: Marín-Padilla, 1990).

**Figure 2:**
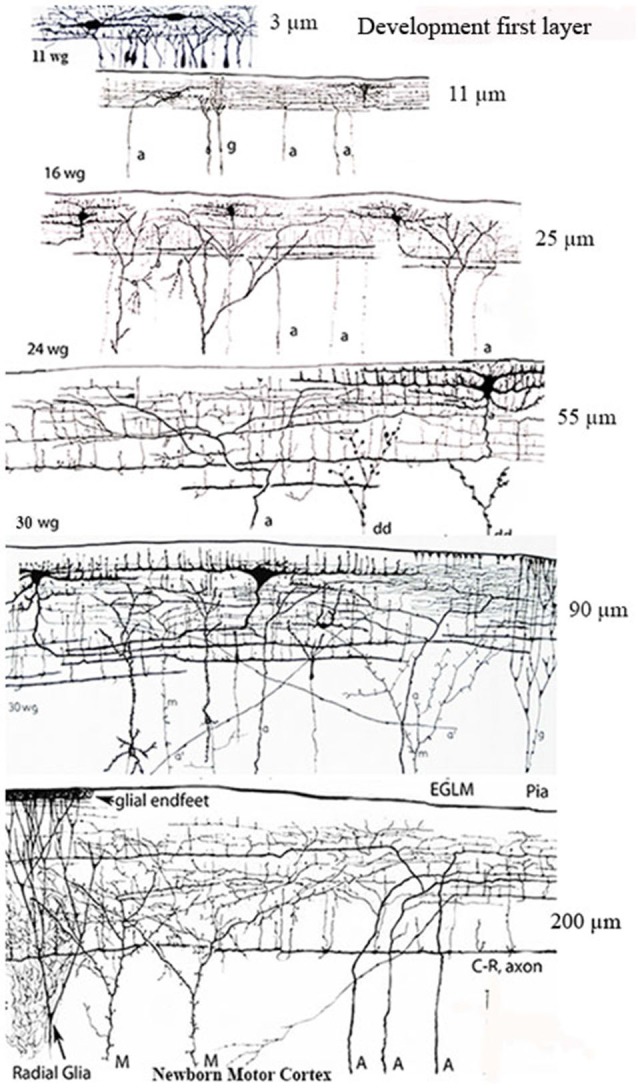
**Camera lucida drawings reproductions of rapid Golgi preparations of the developing human motor cortex showing the progressive structural and functional maturations of the first lamina, of its C-RCs and of its original afferent fibers, through 11-, 16-, 24- and 30-week-old fetuses and newborns infants**. The first lamina increasing thickness is expressed in micrometers on the right of each gestational age. The lamina increasing thickness results from the increasing number of pyramidal cells terminal dendrites reaching and maturing within it. The C-RCs essential morphology remains unchanged during the neocortex entire prenatal development. Original afferent fibers (a, A) from the white matter reach the developing first lamina and become long horizontal terminals through its upper region, where the C-RCs body and dendrites are located. The C-RCs descending axon gives off several thin horizontal axonic collaterals through the lamina middle region and become a long thick horizontal fiber through its lower region. The axonic terminals of Martinotti cells (M) also reach and branch locally within the first lamina. Numerous radial glia fibers reach the first lamina contributing glial endfeet to the neocortex external glial limiting membrane. Later in development, axonic terminals (a’) from thalamic, interhemispheric and cortico-cortical fibers also reach the first lamina and are locally distributed. There are no reasons to think that the extraordinary concentration of fibers terminals within first lamina will disappear in the adult brain since none of its terminal dendrites do. See also Figure 7B. Key: a = afferent fibers terminals; g = radial glial cell terminals; dd = degenerating dendrites often recognized during mid-gestation; M = Martinotti cell axon terminals; C-R = C-RCs axonic terminals; Pia = pial surface. (From: Marín-Padilla, 1990).

**Figure 3:**
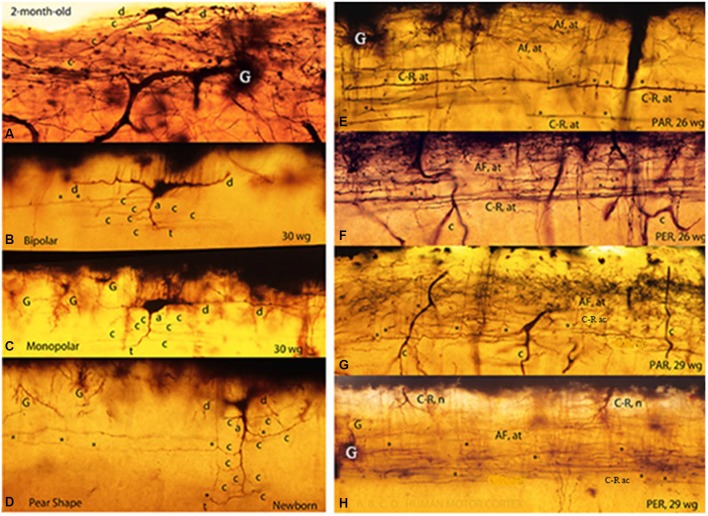
**Composite figure of photomicrographs of rapid Golgi preparations of the human motor cortex’ first lamina showing the C-RC’ body variable morphology, including irregular (A), bipolar (B), monopolar (C) and pear shapes (D)**. The middle location of the neuron thinner axonic collaterals (C-R ac) and the lower one of its thicker axonic terminals (C-R at) are shown in parallel **(E,G)** and perpendicular **(F,H)** cut sections of the motor cortex first lamina from 26- and 29-w-o fetuses. The first lamina structural organization is undistinguishable in parallel and perpendicularly cut preparations. Some C-RC’ bodies **(H)** and the upper distribution of the terminals (AF. at) of original afferent fibers **(E–H)** are also shown. At this age, the first lamina thickness is roughly 90 micrometers (From: Marín-Padilla, 1990).

The neurons original descriptions by Cajal, Retzius and myself coincide in all features (Figure 1). In perpendicular sections, the C-RC’ soma and dendrites are located within the lamina upper region and their morphology may take a pear shape, monopolar and/or bipolar (Figure 1). The neuron descending axon gives off several horizontal collaterals distributed throughout the lamina middle region and terminates into a horizontal axonic fiber that runs through its lower region (Figures 1, 2, 3A–D). During late prenatal development, the number of C-RCs horizontal axonic collaterals increases paralleling the functional expansion of the pyramidal neurons terminal dendrites (Figure 2). Consequently, the first lamina and the C-RCs thickness increase concomitantly (Figure 2). Although, the C-RC essential morphology remains basically unchanged during the cortex subsequent maturation, it will be progressively modified (Marín-Padilla, 2014).

**Figure 4:**
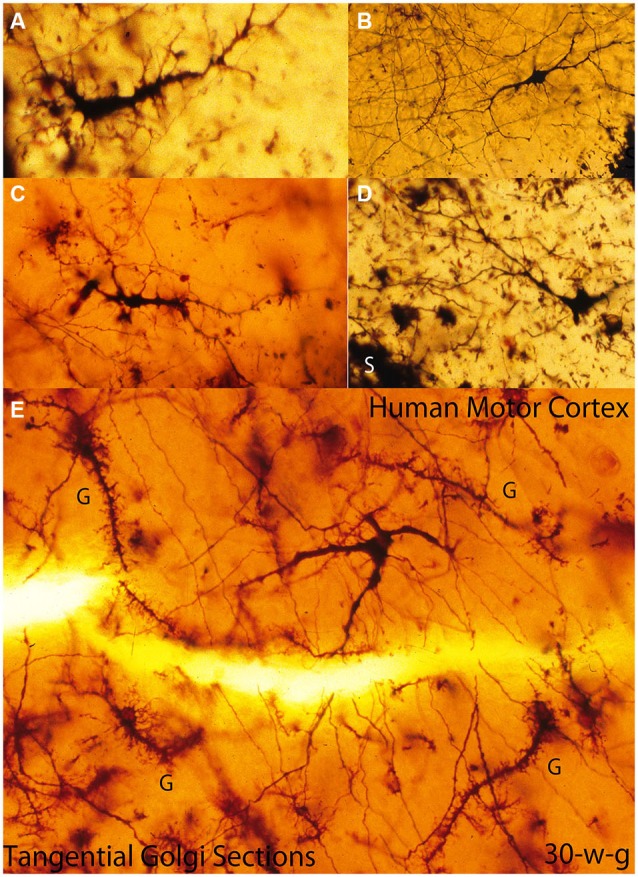
**Composite figure of photomicrographs of tangentially cut Golgi preparations of the motor cortex first lamina of 30-w-o fetuses showing the actual multipolar morphology of C-RCs’ bodies (A-E), the first lamina special glial (g) often referred as comet cells (E) and the crisscrossing of fine axonic terminals (H) through the lamina upper region**. (From: Marín-Padilla, 1990).

**Figure 5:**
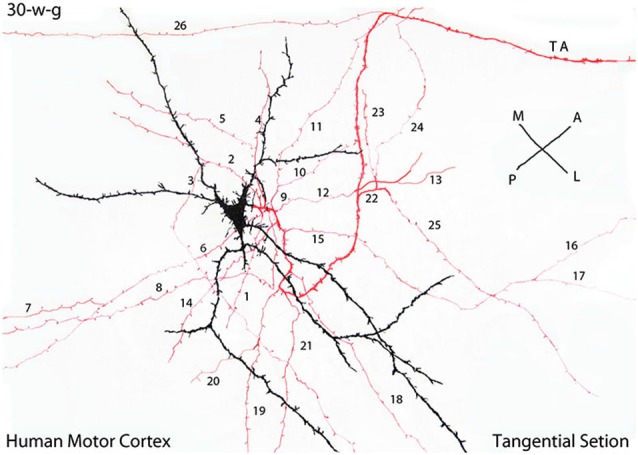
**Camera lucida drawing of a tangentially cut rapid Golgi preparation of the motor cortex of a 30-w-g fetus, illustrating the multipolar morphology of a C-RC with radiating dendrites (black), a descending axon with numerous (26) radiating horizontal collaterals (red) and the neuron axon (TA) terminal (red) that projects in an antero-lateral direction**. The radial expansion of the axonic collaterals of each CRC establishes a functional circle making functional contacts with all pyramidal cells terminal dendrites within it. At 30-w-g, the diameter of the functional territory of this particular C-RC is already 14 mm. Since both the neuron axonic terminals and the pyramidal cells dendrites are already present in the first lamina their functional territory will continue to expand functionally during the neurons subsequent maturation. During cortical development, the diameter of C-RCs functional circles increases progressively, intermingle with neighboring ones eventually covering the entire first lamina and will contact all pyramidal cells terminal dendrites throughout the neocortex. The illustrated rectangular region measures roughly 380 × 150 micrometers. (From: Marín-Padilla, 1990).

**Figure 6:**
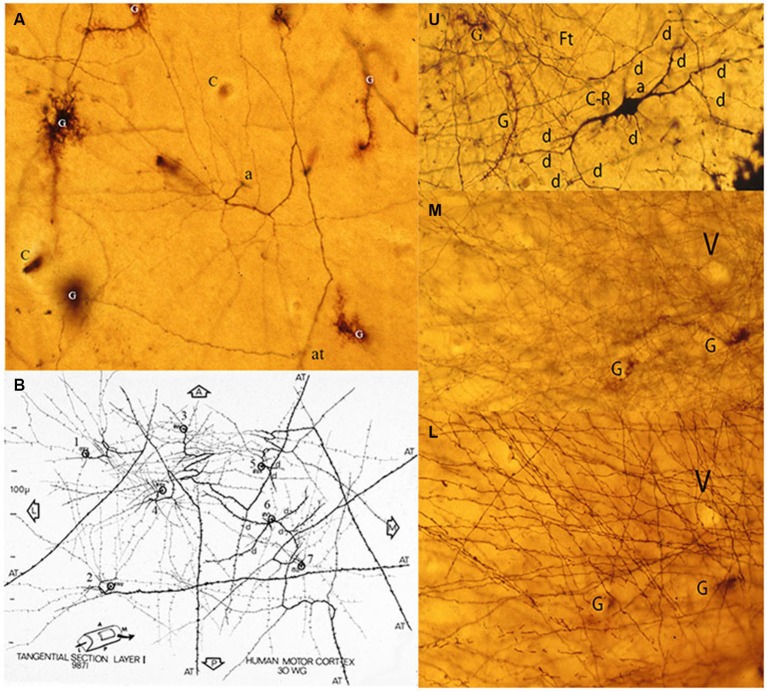
**Composite figure of photomicrographs and a camera lucida drawing (B) of tangentially cut rapid Golgi preparations of the motor cortex of a 30-w-g human fetus**. **(A)** Tangential view of a C-RC axon (a) with numerous radiating collaterals and its axonic (at) terminal, first lamina special astrocytes (comet cells) and some capillaries (c). The neuron’ radiating axonic collaterals establish an expanding functional circle contacting all pyramidal cells terminal dendrites within it. **(B)** Montage of camera lucida drawings of the axons from seven C-RCs with their radiating axonic collaterals and terminal axons projecting anteriorly, posteriorly and/or laterally within the first lamina. U, M, L. Three consecutive tangential views of first lamina, from a 30-w-o fetus, showing in the upper level **(U)** the neuron’ multipolar morphology (d), a descending (a) axon and some astrocytes (g). The middle level **(M)** shows the crisscrossing of the neuron’ fine axonic collaterals and a tangentially cut capillary (v). The lower **(L)** level shows the crisscrossing of the neuron thicker axonic fibers, the same capillary (v) and glial (g) cells. The thickness of a single Golgi preparation (ca. 150 μm) permits to visualize the first lamina structural organization in its entirety (From: Marín-Padilla, 1990).

**Figure 7:**
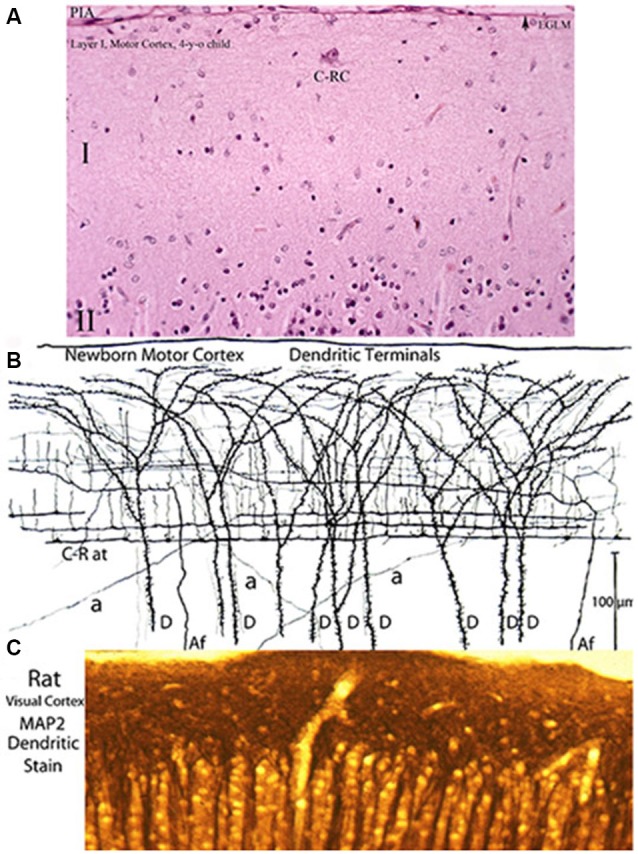
**Three entirely different views of the neocortex first lamina’ composition and structural organization obtained with various staining methods**. **(A)** Reproduces its essentially empty appearance in hematoxylin and eosin preparations (the most commonly used staining procedure) of a 4-year-old child motor cortex showing a large C-RC (C-RC), several small glial cells and, practically, nothing else. The lamina pial surface external glial limiting membrane (arrow) and the upper edge of the second (II) lamina are also illustrated. **(B)** Montage of camera lucida drawings from rapid Golgi preparation of a newborn’ motor cortex showing numerous dendritic and axonic terminals that constitute its essential components. The pyramidal cells terminal dendrites **(D)** represent the first lamina main receptive elements. Most of the fibers are from C-RCs’ axons. Its thicker axonic terminals (C-R at) run through the lamina lower zone and its thinner axonic collaterals through its middle zone. Both axonic terminals have numerous ascending and fewer descending branchlets that represent the neuron’ functional elements. While C-RCs axonic terminals are recognized throughout the first lamina, the neuron’ bodies are quite difficult to locate because of their developmental dilution. During early development, the first lamina also receives afferent fibers (Af) from the white matter that are distributed through the lamina middle and upper zones. During late development, the first lamina also receives additional terminals (a) from thalamic, interhemispheric and cortico-cortical fibers that are distributed locally. **(C)** Reproduces a view an adult rat visual cortex stained with MAP2 (microtubules associated protein-2) a specific stain for dendrites, demonstrating that the concentration of dendrites within the first lamina is by far the greater throughout the neocortex (Donated by Professor Alan Peters, Boston University School of Medicine). These morphological discrepancies about the neocortex first lamina composition and structural organization have also been a source of controversy. Of the three staining procedures, the classic Golgi method conveys the most accurate account of the first lamina composition and structural and functional organizations. The rectangular sections illustrated **(A,B)** measure roughly 230 × 150 micrometers and **(C)** 150 × 60 micrometers. See also Figure 2.

The C-RCs’ axon and its collaterals have numerous ascending and fewer descending thinner branches, considered to represents its functional terminals (Figures 1, 2, 3A–D). Since the terminal dendrites of pyramidal neurons are the only receptive elements within the first lamina they are considered to represent the C-RC functional targets (Marín-Padilla, 1984).

The functional target of the original afferent fibers that reach the first lamina (Cajal, 1911, 1933; Marin-Padilla, 1971) must be the C-RCs since they are the only first lamina neurons during early cortical development. Eventually they could also target the terminal dendrites of arriving pyramidal cells. These afferent fibers terminals intermingle with C-RCs dendrites and with the pyramidal terminal dendrites throughout the first lamina, suggesting a functional interaction among them (Marin-Padilla and Marin-Padilla, 1982; Marín-Padilla, 1984). C-RCs’ evolving morphology and first lamina structural organization, composition and thickness cannot be separated, as they are mutually interdependent (Figure 2).

Retzius, Cajal and myself pointed out that the neuron long horizontal axonic terminals (tangential fibers of Retzius) acquire myelin sheath during early postnatal life. In humans, their myelination starts around the fourth and fifth postnatal years (Kaes, 1907; Langworthy, 1933; Yakolev and Lecours, 1967; Rocker and Riggs, 1969; Retzius, 1894; Brody et al., 1987). They are the earliest components of the gray matter to undergo myelination, emphasizing their functional relevance. Perpendicular sections of myelin stained (Klüver-Barrera method) preparations of children and adult motor cortices show myelinated fibers running through the first lamina lower region (Marín-Padilla, 1990). While in tangential section these myelinated fibers crisscross each other throughout first lamina.

During late prenatal development, small neurons and the terminals of specific thalamic, interhemispheric and cortico-cortical fibers are also incorporated in the first lamina (Marín-Padilla, 1984). While C-RCs’ axonal terminals and those of the afferent fibers could establish contacts with all pyramidal cells terminals dendrites throughout the first lamina, these fiber incorporated later only establish contacts with the terminal dendrites of regional pyramidal cells. These late incorporated fibers are possibly related to the eventual motor, sensory, acoustic, visual and/or associate functions of regional pyramidal neurons.

The C-RCs body variable morphology (upper region), that f its thinner axonic collaterals (middle region) and that of its thicker axonic terminals (lower region) were essentially identical in both parallel and perpendicular cut. It might be a reason that could explain why Golgi preparations cut perpendicular and/or parallel to the precentral gyrus long axis are morphologically undistinguishable (Figures 3E–H). The study of tangentially cut Golgi preparations solve the paradox, roughly 100 years of the neuron original description (Marín-Padilla, 1990).

In tangentially cut Golgi preparations, the C-RC appears as a three-dimensional multipolar neuron with radiating dendrites and a descending axon with long radiating horizontal collaterals and terminates into a long horizontal fiber that may projects in any direction within the cortex (Figures 4, 5, 6A,B). The direction of any perpendicular section of the first lamina will determine the neuron’ body pear shape, monopolar and/or bipolar morphologies (Marín-Padilla, 1990). Moreover, Cajal (1911) described and illustrated a large neuron with radiating dendrites in methylene blue preparation of the cat cortex first lamina as well as the crisscrossing of axonic terminals. He did not realize that it could have represented a tangential view of a C-RC.

Since Golgi preparations thickness range between 150 to 200 micrometers, is possible, in a single tangential cut, to visualize the first lamina entire thickness and structural organization (Figures 6U,M,L). Tangentially cut Golgi preparations of first lamina will show C-RCs bodies with variable morphologies through its upper region (Figure 6U), the crisscrossing of the neuron thinner axonic collaterals through its middle one (Figure 6M) and the crisscrossing of the neuron thicker axonic terminals through its lower one (Figure 6L). The fact that both the C-RCs’ axonic collaterals and terminal axons crisscross each other within the first lamina, during mid gestation, was puzzling and also required clarification. The need to reach all the terminal dendrites throughout the expanding cortical surface will explain the radial expansion of C-RCs axonal collaterals and eventually the crisscrossing with those of neighboring neurons through the first lamina middle region. Similarly, the neurons axonic terminals will eventually crisscross each other through the lamina lower region as they project in all directions.

### C-RC function

Essentially, the C-RCs (including bodies, radial axonal collaterals and terminals axons), the original afferent fibers terminals and the pyramidal neurons terminal dendrites are the only components of the neocortex first lamina during prenatal development. These three elements intermingle with each other throughout the first lamina in both the developing and the adult cerebral cortex (Marin-Padilla and Marin-Padilla, 1982; Marín-Padilla, 1984, 1990). Their anatomical interrelations suggest functional ones as well as.

If pyramidal cells terminal dendrites, within the first lamina, are going to be the C-RC functional targets, a radial spread of its axonic collaterals should be expected. Since pyramidal cells terminal dendrites are incorporated progressively into the first lamina, the C-RCs axonal collaterals must elongate radially to meet them, throughout the neocortex first lamina. By its radiating axonal collaterals, each C-RC establishes a circular functional territory establishing contacts will all terminal dendrites within it (Figures 4E, 5, 6A). In the course of cortical development, each C-RC circular territory enlarges as new terminal dendrites are incorporated into it, and those already present expand functionally (Figures 4A–E, 5, 6A,B). The enlarging circular territories of neighboring CRCs will eventually merge with each other. The convergence of neighboring C-RCs functional territories will explain the crisscrossing of their respective axonal collaterals through the lamina middle region (Figures 6M,L). Hence, all dendritic terminals within the first lamina will be contacted by the radial expansion of the C-RCs’ axonal collaterals.

Similarly, during development the neuron thicker axonic terminals of neighboring C-RCs will eventually crisscross each other throughout the lamina the lower region (Figures 6B,L). Tangential sections of myelin stained (Klüver Barrera stain) preparations of adult brains demonstrate the crisscrossing of myelinated axonal terminals within the first lamina (Marín-Padilla, 1990).

Since neither the C-RCs axonal terminals nor the pyramidal cells terminal dendrites disappear, they will continuously share anatomical and functional interrelationships in both developing as well as adult brains. One possible function of C-RCs might be a functional input shared by all pyramidal cell dendrites throughout the neocortex, regardless of their eventual functional role. The nature of this input remains unknown.

In conclusion, the C-RC evolving morphology and possible function responds to the maturations of the terminal dendrites of all pyramidal neurons throughout the first lamina of the neocortex. In conclusion, the C-RC evolving morphology and possible function are intimately entwined with those of the first lamina and the maturing pyramidal cells terminal dendrites.

### The neocortex first lamina composition and structure

Three completely different views as well as conceptions of the neocortex first lamina composition and structural organization emerge from the use of various staining procedures (Figure 7). An essentially barren structure with very few neurons, scattered glial cells and, practically, nothing else, is evident in most routine staining procedures, such as hematoxylin and eosin (Figure 7A). It is not surprising since these methods failed to stain dendrites and/or axonic terminals that represent the first lamina more distinctive and abundant components. This vision of emptiness of the first lamina’ composition and structure have persisted and consequently it has remained inadequately studied. When dendritic and axonic terminals are stained, using the Golgi method, the actual composition and structural complexity of the neocortex first lamina becomes apparent (Figure 7B). The majority of the axonic terminals within the first lamina are from C-RCs and, to a lesser degree, from original afferent fibers. Both fibers are recognized within the first lamina through the neocortex entire prenatal and postnatal maturations (Figures 2–7B). Moreover, when specific staining for dendrites is used (microtubule-associate protein-2), the concentration of dendrites within the first lamina is by far the greatest found throughout the cerebral cortex. This extraordinary dendritic concentration must reflect its functional relevance (Figure 7C). These different views and resulting conceptions of the neocortex first lamina have persisted and have been an additional source of confusions and controversies.

During late prenatal development, terminals from specific thalamic, interhemispheric and cortico-cortical fibers also reach the first lamina (Marín-Padilla, 1984). Their distribution and hence their functional influence within the neocortex first lamina is essentially regional. The functional targets of these late incorporated afferent terminals are the terminal dendrites of regional pyramidal neurons (Figure 7Ba). These late incorporated afferent fibers must be related to the specific functional activity of pyramidal cell contacted by them.

**Figure 8:**
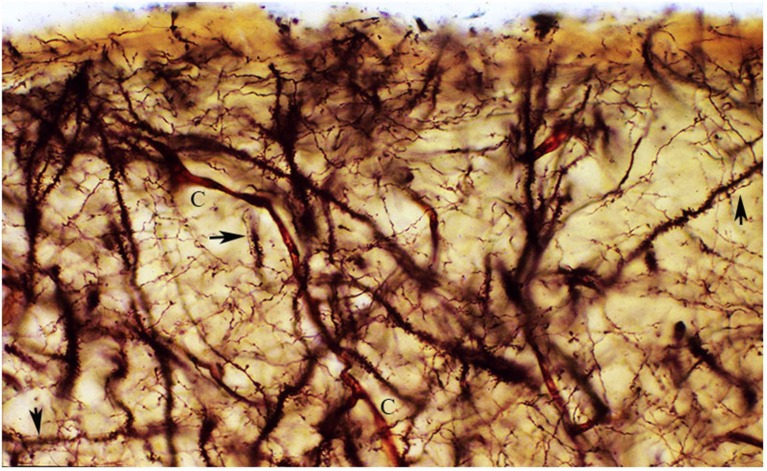
**The figure reproduces a rectangular area of the first lamina upper and middle regions from a newborn motor cortex, measuring roughly 140 × 60 micrometers of a rapid Golgi preparation, the pial surface is at top**. That is essentially composed of axonic and dendritic terminals intermingle with each other. Moreover, synaptic contacts (arrows) between axonic terminals and dendritic spines are often recognized (arrows). To these two elements, blood capillaries (C), special astrocytes and the axonic terminals of the original afferent fibers should be added. Rapid Golgi preparations of the first lamina best and faithfully illustrate its essential components and their structural and functional organizations.

To best visualize and demonstrate the structural and functional complexities of the neocortex first lamina high power microscopic views of Golgi preparations are needed (Figure 8). The rectangular area illustrated in Figure 8, measures roughly 160 × 60 μm and includes the lamina’ upper and middle zones. These preparations demonstrate that the neocortex first lamina is essentially composed of axonic and dendritic terminals that intermingle with each other (Figure 8). Moreover, synaptic contacts between fibers and dendritic spines are often observed in these reparations (Figure 8 arrows). The first lamina structural and functional complexity disclosed in Golgi preparations contract sharply with its apparent emptiness observed with routine staining procedures (Compare Figures 6A, 8).

The functional relevance of the neocortex first lamina is undeniable considering that the terminal dendrites of all pyramidal neurons throughout the neocortex, regardless of size, location or eventual functional role, are represented in it as well as the numerous axonic terminals of C-RCs. The neurons’ soma is difficult to locate because, in the course of neocortical maturation, they have been progressively diluted.

Neither the neocortex first lamina nor the C-RCs functional roles are known. It is time to abandon the controversies and to start investigating the roles they play in overall functional organization of the neocortex. The present account on the structural and functional organizations of the neocortex first lamina and that of its essential neuron, the C-RC, hopefully will stimulate renew interest on both structures, as well as in the need for using the classic Golgi staining procedure.

## Conclusions and future directions

The C-RC is the essential neuron of the neocortex first lamina. It receives inputs from subcortical afferent fibers that reach the first lamina during early in development. The C-RC neuron orchestrates the arrival, size and stratification of all new pyramidal neurons (of ependymal origin) of the neocortex gray matter. Its axonic terminals spread radially and horizontally throughout the entire first lamina establishing contacts with the dendritic terminals of all pyramidal neurons regardless of size, location and/or eventual functional roles. While the neuron axonic terminals spread throughout the entire first lamina targeting the terminal dendrites of pyramidal neurons, its body undergoes progressive developmental “dilution” and it becomes quite difficult to locate any of them in the adult brain. The C-RCs bodies are probably retained in the developing neocortex older cortical regions (such as the primary motor, somatosensory, visual and acoustic regions) while their axonal collaterals will spread throughout its more recent ones (associative areas) that will represent the great majority of the brain surface. This will explain the progressive “dilution” of C-RCs bodies throughout the neocortex.

The human cerebral cortex C-RC is distinguished by distinctive developmental, morphological and possible functional features and by long axonic processes that extend and crisscross throughout the first lamina in both the developing and the adult brains. Therefore, most of the controversies expressed about the C-RCs are not applicable to those in the human cerebral cortex.

Furthermore, the C-RCs’ morphology, nature and possible function cannot be understood without neocortex first lamina where they resided. Their evolutions are concomitant, inseparable and codependent.

The terminal dendrites of all pyramidal neurons of the neocortex gray matter represent the first lamina main receptive elements and the C-RC its main functional unit. The C-RCs axonal terminals reach and contact all pyramidal cells terminal dendrites, within the first lamina, regardless of their size, location and/or eventual functional activity. Moreover, the C-RC terminal axonic branchlets make synaptic contacts with the spines of pyramidal cells terminal dendrites.

The C-RC role within the neocortex overall functional activity remains unknown and should be investigated. It is difficult to comprehend than, more than a century of its discovery we still lack a clear understanding of the C-RC nature and function as well as that of the cortex first lamina. Most controversies about the C-RC and henceforth on the neocortex first lamina have contributed unnecessary confusions and should be left to rest.

Undoubtedly, additional studies are needed to elucidate the C-RC and the neocortex first lamina functions. The following suggestions may help guiding this overdue investigation: (a) the C-RC only known input is from afferent fibers that reach the first lamina early in neocortical development; (b) their origin and function remain unknown and must be among the first ones established in the developing neocortex; (b) it must be necessary to transform all arriving neuroblasts into pyramidal neurons; (c) it must be necessary for orchestrating the arrival, size and eventual stratification of all pyramidal neurons within the gray matter; (d) it must be necessary for maintaining all pyramidal neurons functionally active before they start receiving specific thalamic inputs that will determine their eventual function; (f) it must be limited to the neocortex first lamina; (g) it must reach all pyramidal cells terminal dendrites, within the first lamina, throughout the neocortex; (h) it must be shared by all pyramidal neurons throughout the neocortex, regardless of size, location and/or eventual functional role; (i) it must persists into the adult brain since all pyramidal neurons terminal dendrites also persist; and, (j) it must be distinguished from the specific functional roles of thalamic, interhemispheric and cortico-cortical fibers terminals on pyramidal neurons that are linked to their specific motor, sensory, acoustic, visual and/or associative functions.

By exposing the neocortex first lamina anatomical composition and functional complexity and that of its essential components, namely: the Cajal-Retzius neurons and the dendritic terminals of all pyramidal neurons, this paper hopes to stimulate renewed interest in them as well as in using the Golgi procedure in future brain studies.

## Conflict of interest statement

The author declares that the research was conducted in the absence of any commercial or financial relationships that could be construed as a potential conflict of interest.
